# Optimizing the Detection of Circulating Markers to Aid in Early Lung Cancer Detection

**DOI:** 10.3390/cancers8070061

**Published:** 2016-06-28

**Authors:** Vasudha Murlidhar, Nithya Ramnath, Sunitha Nagrath, Rishindra M. Reddy

**Affiliations:** 1 Department of Chemical Engineering, University of Michigan, Ann Arbor, MI 48109, USA; vasmdhar@umich.edu (V.M.); snagrath@umich.edu (S.N.); 2 Division of Hematology/Oncology, Department of Medicine, University of Michigan, Ann Arbor, MI 48109, USA; nithyar@med.umich.edu; 3 Section of Thoracic Surgery, Department of Surgery, University of Michigan, Taubman Center 2120, 1500 E. Medical Center Drive, Ann Arbor, MI 48109, USA

**Keywords:** biomarkers, circulating tumor cells, microRNA, lung cancer, proteomics

## Abstract

Improving early detection of lung cancer is critical to improving lung cancer survival. Studies have shown that computerized tomography (CT) screening can reduce mortality from lung cancer, but this involves risks of radiation exposure and can identify non-cancer lung nodules that lead to unnecessary interventions for some. There is a critical need to develop alternative, less invasive methods to identify patients who have early-stage lung cancer. The detection of circulating tumor cells (CTCs) are a promising area of research, but current technology is limited by a low yield of CTCs. Alternate studies are investigating circulating nucleic acids and proteins as possible tumor markers. It is critical to develop innovative methods for early lung cancer detection that may include CTCs or other markers that are low-risk and low-cost, yet specific and sensitive, to facilitate improved survival by diagnosing the disease when it is surgically curable.

## 1. Introduction

Early detection of lung cancer, a disease that causes approximately one fifth of all cancer related deaths in the world [1,2], is critical, as early-stage tumors have a five-year survival rate greater than 70% [3]. Most lung cancers are diagnosed when they are too advanced for surgical intervention. The National Lung Screening Trial has shown that early detection of lung cancers results in improved long-term survival. Over 50,000 patients were screened using either low-dose CT scans or chest X-rays. Of patients screened by CT scans, 24% had “positive” scans, with 96% of those found to be false positives. Nonetheless, there was a 20% reduction in lung-cancer-specific mortality and a 7% reduction in all-cause mortality [4]. Despite these improvements, increased radiation from CT scans has been implicated in increasing long-term cancer risk for some, and is not the ideal screening method [5]. Other concerns with the current paradigm of diagnosing lung cancers include the high costs and complications associated with lung biopsies, performed to diagnose indeterminate nodules. Almost 20% of patients can have complications from their lung biopsies [6]. Alternative methods to detect early-stage cancers that can supplant or complement CT screening is an unmet need to increase specificity of screening tests and one that can be more widely used. The identification of circulating biomarkers to detect cancers by blood sampling offers an attractive, less invasive alternative that may eventually change the way we diagnose and treat lung cancer. Here, we review the current state of circulating biomarkers being evaluated for the detection of lung cancers, including circulating tumor cells (CTCs), microRNA (miRNA), circulating tumor DNA (ctDNA), and circulating proteins.

## 2. Circulating Tumor Cells (CTCs)

Circulating tumor cells (CTCs) may be shed from solid organ tumors into the blood stream. They can be separated from normal hematopoietic cells based on size and other inherent physical and biological properties. Recent research has shown that metastasis may not just be a late event, but may be present very early in disease progression [7]. Investigators have demonstrated this in breast cancer using transgenic mice models [7]. They found that tumor cells may be released even before the tumor is presumed to be invasive, and some of these cells may form metastatic tumors. Their findings are supported by reports of tumor spread even after resection of early-stage cancers [7].

In lung cancer, Izbicki et al. studied the presence of micrometastases in lymph nodes and their association with patient survival [8]. In another study, Maruyama et al. analyzed paraffin sections of lymph nodes from stage I patients to predict recurrence from lymph node metastasis [9]. They observed the presence of tumor cells in lymph nodes in 70% of patients, some of whom saw a recurrence [9]. Both studies found a reduction in survival among those patients that tested positive for micrometastatic cancer cells in the lymph nodes. Both of these studies suggest that the presence of these cells indicates that tumor resections might not completely eliminate the disease, and further action such as adjuvant therapy may be needed to detect these “previously undetected” cells [8,9]. The presence of micrometastatic disease in the lymph nodes may also serve as a source for future CTCs found in the blood stream [10]. While there has been debate about the path of cell shedding, namely, whether cancer cells extravasate into the lymph nodes first or the bloodstream first [10], it is now generally believed that undetectable micrometastases may be present before tumor spread to other organs is evident [8,9].

### 2.1. CTCs in Early Cancer Detection

CTC detection in early stages of cancers is gaining momentum as a means of early diagnosis and intervention. A number of studies have demonstrated the presence of CTCs in patients with early-stage breast cancer, with more recent work also being done in lung cancer [11,12,13,14]. Advancing such studies to larger cohorts and detailed molecular analyses of these cells may lead to timely treatment decisions and surgical interventions [14,15]. Kallergi et al. demonstrated the presence of both apoptotic and proliferating CTCs in early- and late-stage breast cancers [11]. The authors observed more apoptotic CTCs in early stages. This could have important clinical implications as CTCs undergoing apoptosis may be used as a surrogate to measure the effect of adjuvant chemotherapy on tumors [11]. Thus, CTC enumeration alone may be insufficient in early stages, and detailed molecular analysis or immunophenotyping is necessary to glean useful information about individual tumors [16]. This could also have potential applications in personalized therapy. In another study of early-stage breast cancer, Nakagawa et al. found correlations between detection of CTCs and lymph node metastasis [12]. Krishnamurthy et al. also demonstrated the presence of CTCs and disseminated tumor cells (DTCs) in early-stage breast cancer, specifically stages T1 and T2 [13]. While the investigation did not reveal significant correlation of CTCs to standard clinical features, they theorize that this could be because of different dissemination mechanisms involved in the metastatic process and that CTCs could still provide useful follow-up and monitoring information [13]. Early-stage breast cancer has provided important insights into the utilization potential of CTCs as an early diagnostic marker, and studies are ongoing in other cancers with the same goal.

### 2.2. CTCs in Lung Cancer

Initial studies characterizing CTCs in early-stage breast cancer set the stage for the study of these blood-based biomarkers in other solid tumors, including lung cancer. Surgical intervention is the most commonly used “cure” for lung cancers when detected early [17]. However, CT scans are limited in their ability to identify very small nodules and predict malignancy of nodules. CTCs or other blood-based markers can complement standard screening tests for early detection [18]. CTCs in early-stage lung cancer have the potential to be interrogated for detailed molecular and genetic characterization.

Ilie et al. used CTCs in conjunction with CT scans to identify high risk patients for having occult lung cancer [14]. Those individuals who tested positive for CTCs despite having no detectable lesions in the CT scan were followed over four years, with all developing CT-scan-identifiable lung cancers that were resected. The study represents a promising direction for future research, wherein CTCs could inform the risk of patients with or even without lung nodules having lung cancer. Ex vivo assays such as the growth of enriched CTCs has also been demonstrated from early-stage lung cancer patients. Zhang et al. captured lung CTCs using a microfluidic post-based device, and expanded them for downstream assays including sequencing [19]. They also showed concurrent mutations of the p53 gene among CTCs and primary tumors in a few samples. Bayarri-Lara and colleagues investigated CTCs in patients undergoing lung tumor resections and found a decrease in the number of CTCs a month after surgery. They also report poorer prognosis in those patients with detectable CTCs despite resection [16]. Wendel et al. studied CTCs from different stages of lung cancer including early stages [20]. While no statistical differences were found between CTC numbers from the various stages, they did observe a high number of CTCs and CTC clusters even in some early-stage patients. This provides scope for further analysis on individual cells and clusters as clusters may carry prognostic information [21]. Supporting this hypothesis, Sawabata et al. observed better survival among patients without CTC clusters during resection [22].

### 2.3. Challenges in CTC Studies

The biggest challenge in the field of circulating tumor cells is posed by their rarity [23]. Overcoming this challenge requires the development of more sensitive technologies that can efficiently pick out these cells from hordes of contaminating cells. Certain factors such as low frequency and low throughputs limit the scope for further CTC analysis [24,25]. Downstream sequencing studies require very a high purity of these cells, and enriched CTCs tend to be either tethered onto the capture device, contaminated with blood cells, or both, thereby increasing noise [25,26,27].

Functional assays such as culturing of these cells require CTCs to be viable and proliferating [28]. The majority of CTCs in circulation are presumed to undergo anoikis or apoptosis, thereby explaining why all disseminated cells do not form secondary tumors. While this poses a big challenge for expanding these cells to perform drug testing and other tumorigenicity assays, it also calls for the use of multiple markers to shed light into why some cells survive physiological shear stresses while others do not [29,30]. Indeed, even apoptotic CTCs may be able to predict prognosis, as shown in the aforementioned study by Kallergi and authors [11].

Another interesting challenge is posed by the theory that these cells may be undergoing various changes in different steps of the metastatic cascade. Epithelial to mesenchymal transition (EMT) is one such change that is believed to be an important part of metastasis, which is mostly attributed to disseminating cells [31,32]. Cell motility and the ability to intravasate into the blood stream is explained by EMT, while the reverse process or mesenchymal to epithelial transition (MET) is believed to occur during extravasation to secondary sites [33]. In light of EMT, there is a general debate about the ability of epithelial markers such as the Epithelial Cell Adhesion Molecule (EpCAM) to capture the wide variety of heterogeneous CTCs [34]. This is currently being overcome by the use of multiple antibodies or by the use of antigen-independent methods of enrichment.

## 3. Circulating RNA

MicroRNAs (miRNAs) are small non-coding single-stranded RNAs of about 22 nucleotides in length. They are transcribed in the nucleus and can be exported by different pathways to the cytoplasm or released into the blood stream. They regulate gene expression post-transcriptionally. They have been shown to be involved in a number of disease processes including cancer [35,36]. Over 2500 miRNAs have been sequenced (miRBASE.org) and have been detected in a wide variety of tissues, including plasma, serum, peripheral blood cells, and urine [36]. Long non-coding RNAs (lncRNA) are highly preserved segments of RNA that have been recently found to both activate and inactivate different genes and, within the context of tumors, can affect oncogenes or tumor suppressor genes [37]. lncRNAs are classified as either small (<200 nucleotides) or long. All RNAs are thought to be stable in the long term within different tissues and are present at detectable levels using current technologies such as RT-qPCR.

### 3.1. RNAs in Cancer Detection

MiRNAs are thought to be differentially expressed in patients with cancer, regulating changes in mRNA expression. Some elevated circulating miRNAs found are related to cancer-specific molecular pathways, but others have been shown to be related to an individual’s immune response to their tumor [36]. With relation to cancer detection, miRNA-21 (miR-21) was first identified in the serum of B-cell lymphoma patients in 2008 [38]. Since then, it has been found to be upregulated in a number of other cancers including prostate, pancreatic, liver, and lung cancers. miR-20a is one of hundreds of miRNAs that has been found to be up- and downregulated in patients with a variety of histologies [39]. Similarly, lncRNAs have been found to be deregulated, with either increased or decreased expression in different tumor types. lncRNAs may offer advantages over other types of circulating markers, as they can be detected at lower concentrations than proteins and may be more tissue-specific than miRNAs [37].

### 3.2. RNAs in Lung Cancer Detection

Current research has shifted from looking at single miRNAs to miRNA combinations, which have been shown to have higher sensitivities and specificities. Bianchi et al. identified a 34-miRNA signature in asymptomatic high risk patients, defined as heavy smokers and over 50 years in age. Their validation set showed 71% sensitivity and 90% specificity, and was lung-cancer-specific, as it was unable to differentiate patients with breast cancer versus benign breast nodules [40]. They further improved the test by reducing their signature to a set of 13-miRNAs and performed validation on a large cohort of patients [41]. Another group has combined a plasma-based miRNA signature with low-dose CT scan screening to reduce the false positive rate from 19.4% in the CT screening group to 3.7% for patients with nodules greater than 5 mm in size [42]. More recently, Wozniak et al. evaluated 754 miRNAs in samples from 100 patients with early-stage non-small cell lung cancer (Stages I–IIIA) and 100 healthy controls. They identified a 24-miRNA panel with the best separation and an AUC of 0.92 [43]. Nadal et al. identified a 4-miRNA signature in serum that could differentiate resectable lung cancer patients from non-cancer patients with an AUC of 0.985 [44]. miRNAs are proving to be an exciting option to identifying early cancer detection signatures. Single miRNAs are unlikely to provide enough discrimination, as multiple signature panels have been found to have an improved ability to identify patients with lung cancer [40,42,43].

### 3.3. Challenges in miRNA Studies

There are numerous technical challenges with working with RNA that precludes its widespread use. miRNAs may originate from the tumors themselves, but levels may also fluctuate secondary to immune responses, and the ability to tell what is tumor-specific is still unclear. microRNA levels may be affected by multiple issues including methods of sampling, RNA stabilization, and the extraction and hemolyzation of samples [43]. Others have suggested that blood cells are the major source of circulating miRNA and that hemolysis can significantly affect miRNA levels [45]. Overcoming these potential causes for inconsistent results are essential before miRNA can be used as a meaningful tool.

## 4. Circulating Proteins

Proteomic approaches to finding blood-based biomarkers have been studied along with CTC and genomic approaches, and was first described in 1996 by Wilkins et al. [46]. Compared to genomic methods, proteomics may be able to more accurately describe the current genetic function of a cell or group of cells, as it includes the post-translational modifications that can occur, as well as account for rates of synthesis and degradation. The proteome may also be able to better reflect the impact that the genetic programming has on the surrounding environment of the cell(s) [47]. There are many ways to identify the array of proteins expressed, including two-dimensional polyacrylamide gel electrophoresis (2D PAGE) and surface-enhanced laser desorption (SELDI) mass spectroscopy. The protein array approach to biomarker identification is not targeted and is similar to the cDNA microarray methods used to identify novel genetic pathways. Alternative protein approaches focus on specific molecules circulating in the blood. C4d is a degradation product of the classical complement pathway. Complement has been shown to be activated in lung cancer cells [48] and is one of many immune responses seen in lung cancer [49]. Monitoring immune activation, as opposed to cancer-specific markers, may allow for the identification of more homogeneous markers, regardless of cancer histology [49]. A more cancer-specific approach is to look for autoantibodies produced to specific tumor proteins. Autoantibodies are created in response to abnormal tumor proteins that may be mutated or misfolded and are recognized by the body as non-autologous [50]. Although autoantibodies are also part of the immune response, they are more tumor-specific than the C4d method.

### 4.1. Circulating Proteins in Cancer Detection

Proteomics has been used to create molecular “panels” in a similar approach to what has been described previously for miRNA. Sohn et al. identified a five-protein signature in patients with triple negative breast cancer that was a predictor of recurrence-free survival [51]. In ovarian cancer, researchers were able to validate a protein panel that was able to stratify patients to long- and short-term survival using an in-depth proteomics analysis of archived patient blood samples [52]. The presence of platelet-C4d has been shown to correlate with vascular embolic events in lupus patients [53], and free C4d has been identified in saliva from patients with oral squamous cell cancers [54]. Individual autoantibodies have been identified in many cancer histologies, including anti-p53 in hepatocellular cancer patients, with an incidence ranging from 12%–73% [50]. Others are studying autoantibody panels, and Kalnina et al. have shown that combinations of autoantibodies can have high specificity (87%–100%) and variable sensitivity (19%–99%) in identifying patients with gastric cancers [55].

### 4.2. Proteins in Lung Cancer Detection

In lung cancer, all three approaches are being investigated with promising early results. Huang et al. used an integrative proteomics approach to identify plasma-based protein biomarkers, specifically dihydrodiol dehydrogenase (DDH) in non-small cell lung cancer patients [56]. Ajona and colleagues have shown that blood C4d was higher in patients with lung cancer than those without [57], suggesting its use in patients with documented lung nodules, but who may not have a diagnosis of lung cancer. Panels of autoantibodies have been validated to identify patients with lung cancer with 93% specificity, although the sensitivities have remained lower at 40% in NCSLC and 55% in SCLC [58]. Chen et al. identified a set of 22 peptides in serum that could predict cancer status with 86% specificity and 85% sensitivity [59]. All methods have shown promising preliminary data, although none are ready for clinical use yet, and there is a lack of validation studies for some of these biomarkers [60].

### 4.3. Challenges in Protein Studies

Proteins offer slightly different challenges than do other biomarkers. The proteome reflects dynamic changes better than other markers, but this then affects consistency in the proteome signature, as proteins are constantly being modified. Protein stability can also affect measurements. 2D PAGE is the most common proteomic approach, but this limits the ability to detect trace levels of proteins, which can limit their use for identifying new markers [60].

## 5. Circulating DNA

Elevated levels of circulating cell free DNA (cfDNA) was first found in cancer patients in 1977 in a study comparing blood samples from 173 cancer patients and 55 healthy controls. Patients with metastatic cancer were found to have higher concentrations than those with localized disease [61]. Since then, more specific circulating tumor DNA (ctDNA) have been identified containing the specific somatic mutations displayed in the primary tumors [62]. ctDNA has been shown to reflect the level of tumor burden and to also reflect dynamic changes in tumor gene expression in response to therapy [63].

### Circulating DNA in Cancer Detection

ctDNA has been identified in patients with a wide variety of cancers, including breast, colorectal, liver, and ovarian cancers [63]. Within lung cancer, current investigations include the evaluation of EGFR mutations identified in ctDNA, and observing tumor response and tumor resistance to EGFR treatments using next-generation sequencing to track ctDNA changes [64]. Newman et al. also demonstrated a novel deep-sequencing approach to study ctDNA in non-small cell lung cancer patients with high sensitivity [65]. Notably, they were able to detect ctDNA from 50% of stage I patients [65], demonstrating applications in early detection. Despite these promising results, there is still limited data supporting the clinical use of ctDNA, as there are a number of technical challenges with ctDNA detection and long-term storage. Dying blood cells can contaminate ctDNA specimens after collection. The heterogeneity of cfDNA and ctDNA has also been shown to complicate the analysis of ctDNA for clinical use [66].

## 6. Conclusions

Circulating tumor cells and other circulating biomarkers hold much promise in the near future as tools for cancer detection. Different approaches have been shown to have different strengths and weaknesses (Table 1), but there are promising specific markers identified ranging from CTC clusters to miRNA panels and immune response related proteins. The ability to diagnose early-stage lung cancer without needing a tissue biopsy or a screening CT scan may soon arrive, which would drastically improve survival rates by increasing the percentage of patients who are diagnosed with operable lung cancers.

## Figures and Tables

**Table 1:** Comparison of different circulating biomarkers.

Marker	Pros	Cons	References
Circulating tumor cells	Can reflect tumor morphology and pathological identification	Very rare	[23,26,67,68,69,70,71]
	Prognostic indicator of disease	Downstream assays may be affected by blood cells	
	May represent tumor heterogeneity		
	Can also be used for genetic analysis such as gene expression profiling or mutation analysis		
Circulating RNA	May provide tissue-specific information	Multiple miRNA signatures required to provide clinical utility	[36,37,40,42,43]
	May provide patient-specific response	Tumor-specific signatures may be indistinguishable from other signals	
		miRNA may be affected by hemolysis	
Circulating proteins	Inform dynamic changes occurring in cell	Constant modification of proteins not reflective of a specific signature	[47,60]
	Inform changes caused to microenvironment	Difficult to detect very low amounts of protein	
Circulating DNA	Monitor treatment response in patients	Dying cells can contaminate samples	[26,66,71,72]
		Difficult to differentiate between cell free DNA and circulating tumor DNA	
